# Statistics anxiety and predictions of exam performance in UK psychology students

**DOI:** 10.1371/journal.pone.0290467

**Published:** 2023-08-23

**Authors:** Benjamin W. Hunt, Tyler Mari, Graeme Knibb, Paul Christiansen, Andrew Jones

**Affiliations:** 1 Psychological Sciences, University of Liverpool, Liverpool, United Kingdom; 2 Psychology, Edge Hill University, Ormskirk, United Kingdom; 3 School of Psychology, Liverpool John Moore’s University, Liverpool, United Kingdom; University of Glasgow, UNITED KINGDOM

## Abstract

**Background:**

Statistics anxiety is common among social science students. Despite much evidence examining statistics anxiety and test performance, little research has explored the role of student self-prediction on test performance in a higher education setting.

**Objective:**

The purpose of this study was to investigate the relationship between statistics anxiety and both students’ self-prediction of their future exam performance and actual test performance on a formal statistics assessment at undergraduate level in psychology students in the UK.

**Method:**

Using a cross-sectional design, two hundred and two students were required to complete Statistics Anxiety Rating Scales, the Mathematical Prerequisites for Psychometrics Scale, and provided self-predicted test performance scores. Test performance data was obtained from a formal statistics assessment.

**Results:**

As predicted, we demonstrated statistics test anxiety to be negatively associated with self-predicted performance. Additionally, we found statistics anxiety was positively associated with test performance.

**Conclusion:**

The findings highlight the complex relationship between statistics anxiety and test performance, suggesting there may be an optimal level of anxiety for performance in statistics assessments.

**Implications:**

The results we report have implications for psychology research methods and statistics instructors who may wish to incorporate the findings into statistics instruction modules in order to assuage high levels of statistics anxiety and foster student well-being.

## Introduction

Statistics anxiety is commonly defined as ‘*extensive worry*, *intrusive thoughts*, *mental disorganisation*, *tension*, *and physiological arousal when exposed to statistics content*, *problems*, *and instructional situations*’ [1]. It is prevalent across various academic disciplines including Psychology, Sociology, Business, and Education Studies [2, 3]. Estimates suggest that more than 70% of university students experience some anxiety when learning statistics [3], and this has led to reports of students deliberately choosing courses which do not contain a statistics learning element.

Psychology undergraduate courses have a considerable statistics component, and the teaching of research methods and statistics is a requirement for these courses within the UK (e.g., for British Psychological Society accreditation) as well as further afield [4, 5]. Evidence suggests that many students are unaware of the considerable statistics component before taking the course. Ruggeri and colleagues [6] observed that only 46% of students were aware of the necessity for statistics in a psychology course and when asked, reported that they ‘*never see why it matters in psychology*’. The lack of awareness of statistics is potentially problematic as statistics anxiety is associated with mental health concerns, and students who have statistics anxiety may increase their risk by taking courses with an unknown statistics element. For example, Macher et al. [7] demonstrated a significant association between statistics anxiety and trait anxiety in a sample of 147 German undergraduate students. Moreover, research has demonstrated a positive correlation between statistics anxiety and depression in a sample of 411 undergraduate students from a university in Bangladesh who enrolled on a statistics course [8]. Similar research suggests that mandatory statistics courses trigger high levels of worry, and have a bad reputation among students [9].

Considerable attention has been given to the impact of statistics anxiety on academic performance. Students have reported actively avoiding statistics, such as skipping the statistics/results section in journal articles [6], which is likely to impact their wider understanding. Some evidence suggests statistics anxiety is negatively related to examination performance [10] and acts as a barrier to learning [3], with delays to completion of the degree programme and even abandonment of studies being reported [11]. However, there is also evidence to suggest there is no significant link between statistics anxiety and examination performance [12]. The overall evidence is equivocal and a recent review concluded that the inconclusive evidence may be a consequence of cognitive and individual differences (e.g., procrastination: [13]). Nevertheless, it is plausible that statistics anxiety contributes to reduced academic performance, but the relationship may not be simple, linear, or constant over time [10, 14]; warranting further investigation.

A further, domain related, aspect of statistics anxiety is self-perceived mathematical ability [15]. Previous research has found that the two constructs are highly, negatively correlated [11]. Understandably, mathematics has an anxiety related construct of its own, with similarities and differences when compared with statistics anxiety [16]. Given statistics anxiety’s relationship with both self-perceived mathematical ability and mathematics anxiety [17], it seems prudent to include an objective measure of mathematical ability as a control in analyses that explore the role of statistics anxiety in an educational setting.

Perhaps surprisingly, statistics anxiety has also been proposed to aid students in achieving optimal examination performance, possibly due to the Yerkes-Dodson law which states that a modest level of arousal has a beneficial effect on performance [18]. Indeed, Keeley and colleagues [14] examined the relationship between test anxiety and test attainment in a sample of 83 social science students. The results demonstrated a quadratic relationship between test anxiety and performance, with individuals reporting low and high anxiety scores exhibiting lower test scores than those with a moderate level of anxiety. The results suggest that there may be an optimal level of statistics anxiety, which may contribute to improved examination performance. However, given the heterogeneous findings between statistics anxiety and exam performance, additional exploration of the relationship remains imperative.

The extent to which students are able to predict their performance on a future statistics assessment may be a valuable avenue for exploring the underlying mechanisms of statistics anxiety. Beyond examination performance, statistics anxiety may also be associated with students’ self-perceived ability. For example, research has shown that statistics anxiety is negatively associated with students’ self-predicted grades, a finding which led to the authors’ suggestion that students are often unaware of their own statistical abilities [19]. However, research exploring the association between statistics anxiety and student self-predicted outcomes is sparse. Consequently, further research is required to better understand the nature of the relationship. Therefore, the aim of this study was to examine the role of statistics anxiety on students’ prediction of their future exam performance, as well as their actual performance on these exams, in a group of UK undergraduates. We hypothesised that statistics anxiety would be negatively associated with students’ self-predicted examination performance. Given the equivocal literature, we made no directional hypotheses regarding the relationship between statistics anxiety and actual examination performance.

## Method

### Participants

Three hundred and twelve participants accessed the survey, with 214 completing it. Of these, we were unable to obtain marks for nine individuals and three individuals reported either non-binary/ preferred not to disclose their gender. We were unable to create a meaningful analysis group based on this, therefore this left us with an analysis sample of 202 participants. The demographic information of these participants is shown in Table 1. To summarise, they were generally representative of UK undergraduate psychology courses in that the majority of students started at age 18 and were female. Inclusion criteria included enrolment as a first-year undergraduate psychology student at the University, which required the completion of two taught statistics models (one in each semester). There were no exclusion criteria. Regarding the chosen sample size, our power analysis was based on an R^2^ of .10 for the summative models, indicative of a small-to-moderate amount of variance explained in our outcome variables. Our rationale for this was the small-moderate correlations between statistics anxiety and performance as reported throughout the literature, as well as a large amount of variance (37%) explained in estimated exam scores by statistics anxiety in an analysis conducted by Hanna and Dempster (2009). We opted for 80% power and alpha < .05. This required 172 participants, based on 11 predictors (described in analysis section). The research was approved by the Local Research Ethics Committee (date: 26-08-2020; Ref: 8075).

**Table 1:** Demographic information of the sample of UK undergraduates.

Variable	N	Mean (SD)	Min	Max	Scale scores (range)
Age	202	18.51 (0.84)	18	26	
SA: Fear of asking for help	201	10.01 (4.05)	4	20	4–20
SA: Fear of statistics teacher	199	9.61 (3.61)	5	25	5–25
SA: Worth of statistics	199	33.95 (11.00)	16	62	16–80
SA: Self-concept	200	18.30 (6.67)	7	35	7–35
SA: Interpretation anxiety	202	32.04 (7.35)	16	54	11–55
SA: Test anxiety	201	23.41 (5.19)	9	35	8–40
MPPS score	202	10.98 (2.64)	2	15	
Predicted exam grade	202	64.03 (7.44)	20	100	
Predicted rank	202	40.64 (14.60)	10	90	
Confidence in prediction	199	52.00 (18.57)	1	86	
Actual grade	202	75.02 (16.37)	0.00	96.91	
**Variable**	**N**	**%**			
*Gender*					
Females	155	76.7			
Males	47	23.2			

MPPS = Mathematical Prerequisites for Psychometrics Scale; SA = statistics anxiety

## Measures

### Statistics Anxiety Rating Scale (STARS: Cruise et al. [20])

The STARS is a widely used measure of statistics anxiety and attitudes towards statistics. It consists of 51 items which load onto 6 distinct subscales [21]; test and class anxiety (e.g. ‘*Doing an examination in a statistics course’*), interpretation anxiety (e.g. ‘*Interpreting the meaning of a table in a journal article*’), fear of asking for help (e.g. ‘*Asking a fellow student for help understanding a printout’*), worth of statistics (e.g. *‘Statistics takes more time than it is worth’*), fear of statistics teachers (e.g. ‘*Statistics teachers are so abstract they seem inhuman’*), and computational self-concept (e.g. *‘I do not have enough brains to get through statistics’*). Participants rate each item using a 5-point Likert scale. For the anxiety-related subscale questions (test and class anxiety, interpretation anxiety and fear of asking for help) the response anchors are 1 (no anxiety) to 5 (very much anxiety). For the attitude-related subscale (worth of statistics, fear of statistics teachers, computational self-concept) questions, the response anchors were 1 (disagree) to 5 (agree). Although other examples of statistics anxiety measures exist (e.g., [22]), the STARS measure was chosen as it is the most widely used measure of statistics anxiety and previous research has demonstrated its validity in English speaking undergraduate populations ([21, 23]), thus allowing direct comparisons with our sample. Here we observed adequate internal consistency for all subscales: test and class anxiety (Omega = .85), interpretation anxiety (Omega = .89), fear of asking for help (Omega = .92), worth of statistics (Omega = .93), fear of statistics teacher (Omega = .81), and computational self-concept (Omega = .81).

### Mathematical Prerequisites for Psychometrics Scale (MPPS: Galli et al., [24])

The MPPS was designed to examine the mathematical-related abilities that psychology students will require for introductory statistics courses, such as relations, fractions, and probability. It contains 30 multiple-choice questions, with four answers (one correct), for example ‘*If I choose numbers 13 and 17 in a lotto game*, *which of the following numbers has a higher probability of being drawn*?’ [13 or 17; 13 and 17; 13; 17]. Previous research has demonstrated that items load onto one scale [24], therefore, to reduce the length of the study we used only 15 items. The outcome variable was the number of correct answers.

### Predicted grade, predicted rank, and actual exam performance

Participants provided predictions of their likely grade (0–100%) and where this would rank them relative to the class (cohort) (bottom 0% to top 100%). Confidence in prediction of grade (0 [not confident at all] - 100 [extremely confident]) was also provided. Actual Exam Performance data was obtained after participants undertook the final (end of semester) statistics exams in January, 2021 and June, 2021. The marks from the two exams was averaged into a total score for each participant. These three variables were employed as dependent variables in the analyses.

### Procedure

In the first two weeks of the first semester (October, 2020), first-year undergraduate psychology students at a large UK university responded to an advertisement for a study on “Statistics and Exam Stress.” Individuals who indicated an interest in participating in the study were sent a link to complete the questionnaires online (via Qualtrics). Participants accessed the link and provided informed consent. They were then asked basic demographic information (age, gender [male, female, non-binary, prefer not to say]). Next, participants were asked to provide some identifying information such as name or student number to allow us to match up their grades at the end of the two semesters in that academic year (the mark from each semester was averaged into a total mark). This enabled identification of individual participants to members of the research team (the authors). They were then asked to provide predictions of their grade in the end of year (final) exam, where this would rank them in the class, and how confident they were in their prediction. Lastly, participants completed the STARS and the MPPS. These two questionnaires were presented randomly between participants (i.e., the two measures were counterbalanced) to account for respondent fatigue. Scores on the final statistics exam were obtained at the end of the same academic year.

### Analysis strategy

We conducted hierarchical linear regression models to examine the role of statistics anxiety on both predicted grades and actual grades, as this strategy was deemed appropriate for a cross-sectional design. Additionally, these models are often robust to assumption violations [25, 26]. Analyses were performed using the ‘robust’ [27] package in R, version 4.2.0 [28]. For both models, we included gender and age as demographic predictors. We added scores on the short MPPS as a measure of objective statistics knowledge (objective performance step). In addition, this acted to adjust for the potentially confounding presence of mathematical ability. We also added the six subscales of the STARS questionnaire. In the actual grades model, we added their predicted grades and confidence in their predictions. For the predicted grades model, we replicated the first model using their prediction of where they would rank in the class as the outcome variable. We did this as a sensitivity analysis, but we also reasoned as these were first-year university students, they might be less familiar with university grading systems compared to higher education systems (e.g. lettering grades such as A, B, C). We used alpha values < .05 for regression coefficients, as well as overall models, as evidence for statistical significance.

## Results

The Statistics Anxiety subscale scores were broadly similar to those published in a review of undergraduate psychology students [23], see Table 1.

A series of independent samples T-tests found males and females differed on scores on the interpretation subscale (Males = 28.13, Females = 33.23: t(90.31) = 4.80, p = .006: d = .76); test subscale (Males = 20.98, Females = 24.23: t(85.92) = 4.18, p < .001, d = .67) and self-concept subscale (Males = 16.62, Females = 18.78: t(76.34) = 2.01, p = .048, d = .33); but not on any other predictor variables.

### Predicted exam performance

The final model was significant (F(9, 183) = 2.67, p = .006), predicting approximately 5% of variance in the predicted exam performance (Adjusted R^2^ = .05). The only significant predictor of predicted exam performance was test anxiety, which had a negative association (b = -.32 [95% CI: -.07, -.55], p = .010). See Table 2. There were no issues with multicollinearity (Variance Inflation Factors < 2.23).

**Table 2:** Regression models for predicted grade, predicted rank, and actual grade.

	Predicted Grade b (95% CI)	Predicted Rank b (95% CI)	Actual Grade b (95% CI)
Age	-0.853	0.636	-0.036
	(-1.73; 0.02)	(-1.99; 3.26)	(-3.07; 3.00)
Gender (male ref)	0.143	-1.452	-8.88*
	(-2.13; 2.41)	(-6.84; 3.93)	(-16.50; -0.78)
SA: Fear of asking for help	-0.050	-0.187	-0.348
	(-0.35; 0.25)	(-0.80; 0.43)	(-1.10; 0.38)
SA: Fear of statistics teacher	0.081	-0.054	0.062
	(-0.32; 0.48)	(-0.83; 0.72)	(-0.83; 0.93)
SA: Worth of statistics	0.018	-0.246	-0.042
	(-0.11; 0.15)	(-0.49; -0.01)*	(-0.37; 0.27)
SA: Interpretation anxiety	-0.063	0.270	0.030
	(-0.23; 0.10)	(-0.09; 0.63)	(-0.37; 0.43)
SA: Test anxiety	-0.315*	0.121	0.725*
	(-0.55; -0.08)	(-0.40; -0.64)	(0.05; 1.40)
SA: Self-concept	-0.092	0.697*	-0.216
	(-0.29; 0.10)	(0.28; 1.12)	(-0.83; 0.40)
MPPS score	-0.059	-0.582	0.632
	(-0.42; 0.30)	(-1.43; 0.26)	(-0.20; 1.50)
Predicted exam grade			0.092
			(-0.27; 0.46)
Confidence in prediction			0.069
			(-0.06; 0.20)

* p < 0.05.

The model predicting class rank was also significant (F(9, 183) = 3.96, p < .001), predicting 11% of variance (Adjusted R^2^ = .11). Self-concept had a positive association (b = .70 [95% CI: .28, 1.12], p < .001) and self-worth had a negative association (b = -.25 [95% CI: -.49, -.01]. See Table 2. There were no issues with multicollinearity (Variance Inflation Factors < 2.21).

### Actual exam performance

The final model was significant (F(11,178) = 3.34, p < .001), predicting approximately 6% of variance in actual exam performance (Adjusted R^2^ = .06). Males had significantly worse exam performance than Females (b = -8.64 [95% CI: -16.50, -0.78], p = .031). Test anxiety positively predicted exam performance (b = .73 [95% CI: .05, 1.40], p = .034). See Table 2. There was some evidence of multicollinearity for self-concept (VIF = 4.85). When removing this predictor from the model, gender and test anxiety remained predictors but MPPS score was also a significant positive predictor (b = .77 [95% CI: .01, 1.43], p = .048). Note, these findings were robust when using class rank (rather than raw prediction) in the models, but when prediction and confidence were removed from the models the effect of test anxiety became non-significant (b = .60 [95% CI: -.04, 1.25], p = .066].

## Discussion

The findings from this study demonstrated that, in a sample of UK undergraduate psychology students, statistics test anxiety was robustly negatively associated with the predicted exam performance by the end of the year. However, this did not translate into actual performance, with statistics test anxiety positively predicting actual test performance.

Statistics test anxiety was found to be significantly, negatively associated with self-predicted exam performance, as expected. Our findings replicate those of Hanna and Dempster [19] who reported a significant, negative relationship between students’ estimated exam scores and statistics test anxiety in a sample of 52 Irish first-year undergraduate psychology students. Intuitively, student self-prediction might be inversely related to test anxiety due to students’ confidence in their statistical abilities. Indeed, the authors of the aforementioned study found students were unable to accurately estimate their own future exam scores, concluding statistics anxiety to be more closely related to perception of ability than actual performance. A secondary finding from the present study supports this stance, as we observed computational self-concept to be significantly, positively associated with self-predicted class rank, albeit, not with self-predicted grade, nor with performance in the actual exam. This finding is consistent with those also reported by Hanna and Dempster [19] who found this effect in a closely related sample, although, their outcome was self-predicted grade. However, it should be noted that despite the significant finding between test anxiety and self-prediction, the model predicted only 5% of variance and thus may have little ‘real-world’ relevance.

Somewhat unexpectedly, we found statistics test anxiety to be significantly, positively associated with actual exam performance. One possible explanation is that there may be an optimal level of anxiety that aids performance on statistics assessments. Results from the current study may therefore be indirectly supportive of findings from research into optimal statistics anxiety, such as that by Keeley et al. [14] as we found increased levels of anxiety to be related to higher actual performance. A question that arises due to the findings of the present study is what might be the optimal level of statistics anxiety required for successful performance in a formal statistics assessment? Whilst the current study was not equipped to explore this question, future work might focus on optimal or harmful levels of statistics anxiety in relation to test performance. Nevertheless, a similar relationship is demonstrated elsewhere; Macher et al. [11] reported a small but significant direct, positive relationship between statistics anxiety and academic performance. A recent review and meta-analysis conducted by Trassi et al. [13] highlighted the mixed nature of the relationship between statistics anxiety and exam performance concluding that a series of cognitive and individual factors may be able to explain this conflicting evidence, such as procrastination and self-efficacy, as individual attitudes and learning styles (e.g., goal setting, self-monitoring [29]) are likely to play a role in the reduction of anxiety and promoting of test performance.

Few related studies have assessed students’ self-predictions of their future performance in statistics assessments, therefore the inclusion of this under-researched factor’s influence on statistics anxiety and exam performance was a strength of the present study. Given the findings we have reported, student self-prediction may prove to be a useful variable in research that aims to explain the inconsistent relationship previously reported between statistics anxiety and actual exam performance.

There are several limitations associated with the study. As the sample was recruited from the student population of a single university, it is unclear whether the observed effect can be generalised to the wider student population within the UK or further afield, and therefore has limited generalisability. Future research might incorporate student participants from a range of educational institutions and academic disciplines and from a variety of cultural backgrounds. Our measures of statistics anxiety were taken within the first two weeks of study, and it is possible, and perhaps reasonable, that subsequent engagement with course materials on statistics reduces anxiety (see Keeley et al. [14]), and those with greatest statistics anxiety may drop-out. Future research should examine whether changes in statistics anxiety are a better predictor of academic outcomes.

Despite the STARS receiving criticism for measuring constructs other than statistics anxiety (e.g., attitudes towards statistics) and being time consuming for participants (e.g., [30]), we chose to employ this measure due to its validity, and wide-spread use in similar populations which allows for direct comparisons. A future study might benefit from a comparison of multiple statistics anxiety measurements, which could contribute to the reliability of any findings.

Finally, we measured participants’ predicted grade, predicted rank, and scores on the STARS at only one time point prior to obtaining actual test performance results, which represents a limitation of this study. Future work might choose to administer these measures at both baseline and towards the end of the course.

One implication that has arisen as a result of this study is that despite students reporting the presence of statistics test anxiety, the data showed that this may have had a slightly positive impact on actual performance. This finding, therefore, might be usefully incorporated into educational modules to assuage high levels of anxiety and improve well-being in student populations. Given the significant relationship between self-predicted exam performance and test anxiety, one practical implication of the current study might be to screen students prior to beginning a statistics course in order to identify those with lower self-predicted grades and recommend these students undertake an intervention (see [16] for a review of statistics anxiety interventions).

In summary, the present study found statistics anxiety to be significantly, positively associated with actual exam performance, and significantly negatively associated with self-predicted grade, and significant associations with self-predicted class rank. Our findings suggest that students’ confidence in their own abilities plays a role in statistics anxiety, but that experiencing statistics anxiety early in a Psychology course does not negatively impact academic performance, despite predictions to the contrary.
